# Functional Outcome and Complications in Management of Proximal Humerus Fractures Operated with Proximal Humerus Locking Plate

**DOI:** 10.5704/MOJ.2107.008

**Published:** 2021-07

**Authors:** PK George, B Dasgupta, SM Bhaladhare, BPV Reddy, A Jain, AD Jogani

**Affiliations:** Department of Orthopaedics, King Edward (VII) Memorial Hospital and Seth Gordhandas Sunderdas Medical College, Mumbai India

**Keywords:** proximal humerus locking plating, proximal humerus fracture, humerus fracture, PHILOS, humerus plating

## Abstract

**Introduction:**

Controversies exist in treatment of proximal humerus fractures as treatment options vary greatly from conservative management, closed pinning, stacked intramedullary nails, plating and hemi-arthroplasty. The purpose of this study is to study the fracture patterns of each case and document the functional outcome and complications post-operative in the management of proximal humerus fractures operated with proximal humerus plate.

**Material and Methods:**

Thirty five patients with closed proximal humerus fractures, above 18 years old, admitted in our tertiary care hospital during the study period were enrolled. Patients underwent open reduction internal fixation with proximal humerus locking plate under general anaesthesia. Post-operative patients were assessed using Constant and DASH scores. Complications were recorded.

**Results:**

In our study the absolute Constant score of the study population increases at three months and six months and was found to be significant. Mean Constant score for 4-part fractures was 45.6 which were inferior as compared to 2-part and 3-part fractures (43.1 and 44.6, respectively). The mean Constant score at six months was 51.80 +/- 6.71. All three types of proximal humerus fractures showed significant improvement in the mean DASH score over our study period of six months and was found to be significant. Mean DASH score at six months was 27.97+/-12.84. Out of the 35 cases in the study two had complications. One had implant failure (Neer’s type 3, 60-year-old female) and one had varus collapse (Neer’s type 3, 45-year-old male).

**Conclusion:**

Due to angular stability and effective maintenance of the intraoperative fracture reduction during follow-up period, early post-operative mobilisation is possible which helps the patient to attain better shoulder range of motion and return to activity faster.

## Introduction

Fractures of the proximal humerus are complex injuries with significant morbidity. After distal end radius fractures and hip related fractures, proximal humerus fractures are the most common fractures in the elderly population. They represent 5% of all extremity fractures^1,2^. According to the literature the incidence in total population is 70/100000 per annum but over 70 years old, it is 400/100000 per annum^3^. Three fourth of the fractures occur in older population with male to female ratio 1:3. In older population this type of fracture is usually due to minor trauma over a pre-existing osteoporotic bone, whereas in younger population it is often due to high energy trauma.

Treatment options vary greatly from conservative management, closed pinning, stacked intramedullary nails, plating and hemi-arthroplasty. Fractures which are minimally displaced or simple 2 part fractures can be conservatively managed^4^, whereas displaced fractures of 2 or more parts needs to be surgically fixed for better functional outcome. The age of the patient, physical activity and medical fitness also influence treatment choice. A review of current results shows that there is no universally accepted form of treatment. Conservative management may result in non-union, mal-union, avascular necrosis and a painful joint^4,5^.

Treatment protocol was decided by bone quality, fracture pattern, degree of comminution as well as patient factors such as age and activity level and demand of the patient. Final goal of treatment was minimum shoulder pain and maximum range of motion. Surgical options include closed reduction and percutaneous Kirschner pinning, transosseous suture fixation, open reduction and internal fixation with either conventional or locking plate and hemi-arthroplasty. However each fracture should be evaluated separately and decision taken as per fracture pattern.

Thirteen to 16% of proximal humeral fractures consist of 3 or 4 part fractures. Treatment options for these displaced fractures include open reduction and fixation. Neer recommended open reduction and internal fixation for displaced two and three parts fractures^2^. In a three or four part fracture dislocation when the head of the humerus is entirely devoid of any blood supply shoulder arthroplasty is an option.

Now with the era of the locking-compression plate there are promising results for displaced osteoporotic proximal humeral fractures^6,7^. The mechanical advantage of a locking compression plate is that it improves fracture stability due to the fixed-angle construct in which there is no movement between individual parts resulting in an increased resistance to pull-out. Hence, the locking of the screw to the plate mechanically recreates a point of cortical bone contact, which may be useful in poor-quality cancellous bone of the proximal humerus^8^.

Restoration of anatomy, stable fixation, with minimal soft tissue injury preserving the vascular supply is the principle. Locking compression plating overcame some drawbacks of conventional plating techniques, complications have been reported, including avascular necrosis of the humeral head, screw cut-out, head collapse, plate impingement, implant failure and infection^9,10,11^. The complications generally increase with the complexity of the fracture pattern.

This study was to study the fracture patterns of each, document the functional outcome and post op complications as well as discuss the advantages and disadvantages of using Proximal Humerus Locking Plate.

## Materials and Methods

Source of the data included all patients fulfilling the inclusion criteria admitted in our tertiary care hospital during the study period. Inclusion criteria includes acute fracture, age above 18 years old, patients operated using the locking plate system.

Exclusion criteria includes associated same side humerus shaft or distal humerus or the elbow joint fracture, associated neurovascular injury, open fractures, pathological fractures or re-fractures, old fractures or pseudoarthrosis, polytrauma with head injury, pre-existing medical co-morbidities which may hamper fracture and surgical wound healing.

Patients with proximal humerus fractures were selected in a consecutive manner. Clinical and radiological evaluation were done. Fractures were classified using Neer’s classification. Patients underwent open reduction internal fixation with locking plate under general anaesthesia. Postoperative physiotherapy was given. Physiotherapy protocol started with Pendulum exercises for 2 weeks followed by Active Assisted Movements till 12 weeks and then followed by active movements and strength training.

Sample size for this study was calculated using “onesamplemeans” option in Proc Power procedure in SAS. Single sample T-test was applied and normality was assumed. Mean estimate and standard deviation for ROM was used from “Functional Outcomes Following Locking Plate Fixation of Complex Proximal Humeral Fractures by Tingjun Ye; Lei Wang; Chengyu Zhuang, Yazi Wang *et al*;”^12^ research paper. In the current study we used ROM score as one of the primary variables. From the research paper mentioned above, mean ± SD estimate for ROM was found to be 28.1 ± 5.37. The population mean was assumed to be 25.3 and type I error rate (alpha) = 5%. Assuming a power of 80% and using the above estimates a sample size of 31 was calculated. Assuming a 10% dropout over a study the period a sample size of 35 patients was required (N=35).

The surgery was performed in Beach-chair position. Deltopectoral approach was taken. The fractured fragments were reduced in anatomical position and fixed temporarily with 1.5mm - 2mm Kirschner wires. The plate positioned just below the greater tubercle and behind the long biceps tendon. A 3.5mm locking screws of appropriate length were inserted into the head of the humerus and distal fixation to the shaft of humerus done through LCP combi-holes using cortical screws or locking screws depending on the quality of the bone as shown in Fig. 2. The reduction was confirmed under C-arm. Sterile wash given and wound closed in layers and sterile dressing given. Post-operatively patient was assessed clinico-radiologically, range of motion noted and graded according to Constant Shoulder Score^13^ and the Disabilities of the Arm, Shoulder and Hand (DASH) score^14,15^.

**Fig. 1:**
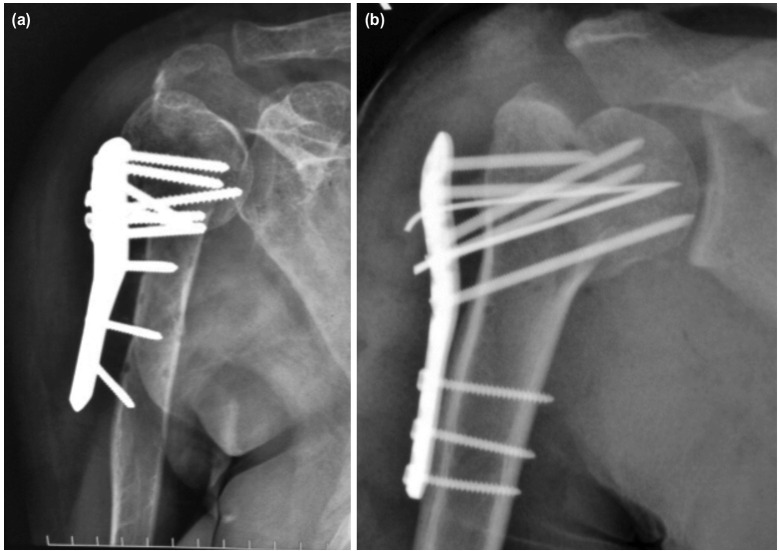
(a, b) Incidence of complications in the study population.

**Fig. 2:**
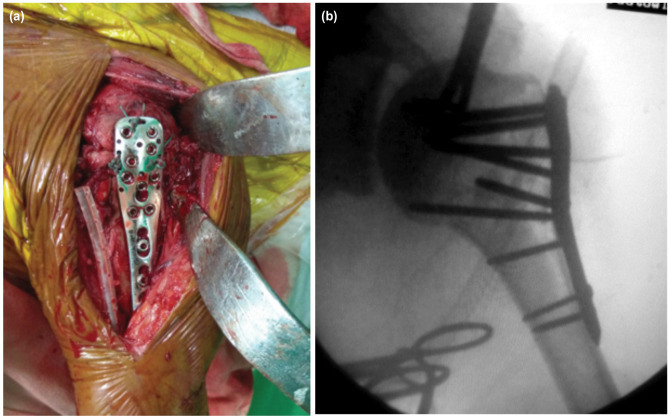
(a, b) Plate position and intra-operative C-Arm.

## Results

Patients were grouped into four age groups 18-30, 31-50, 50-70, more than 70. The age distribution of the study population was from 25 to 82 years with a mean age of 52.3 years. The highest incidence of proximal humerus fractures was found in the age group of 31 to 50 years accounting for 48.6% (17 out of 35) of the study population.

Out of the 35 cases 25(71.43%) were male and 10 (28.5%) were female. The ratio between males and females was 2.5:1. The incidence of fracture was found to be more on the right (dominant) side (22 out of 35; 62.86%) compared to the left (non-dominant) side (13 out of 35; 37.14%).

The most common mechanism of injury was due to road traffic accident (48.57%) followed by fall (45.71%) followed by fall over the involved shoulder due to seizure (5.71%). The incidence of road traffic accidents was found to be more common in males (12 out of 17 cases). The incidence of fracture due to fall was more common in the elderly age group while road traffic accidents was more common in the young though the difference was not significant (p-value > 0.05)

**Fig. 3:**
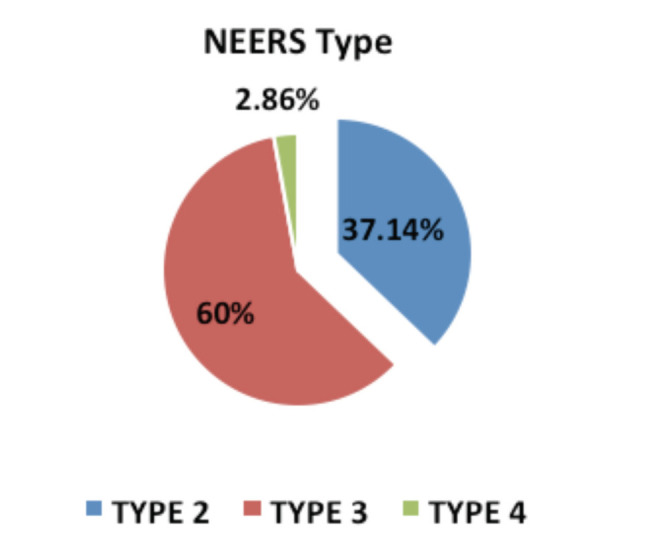
Distribution of the Neers pattern in the study population.

Neer’s Type-3 fracture pattern was found to be the commonest in our study population accounting for 60% (21 out of 35) of the cases whereas type-2 fracture was seen in 37.14% (13 out of 35) of the cases and type-4 in 2.86% (1 out of 35) cases as shown in Fig. 3. Type 2 was found to be more common due to fall (9 out of 13) whereas Type 3 and 4 was found to be more common due to high energy trauma due to road traffic accidents (14 out of 22). In our study 5 out of 35 patients (14.29%) required primary bone grafting. All 5 were above 70 years of age.

The range of motion was evaluated and the following observations were obtained. At one and half months 62.8% (22 out of 35) of patients had flexion < 60. At 3 months 88% (31 out of 35) in the range 60-90. At 6 months 60% (21 out of 35) in the range 90-120 as shown in Table I. Thus we have found that flexion improved over the study duration and was found to be significant (P value <0.001 Fisher’s exact test used)

**Table I:** Number of patients in defined flexion range at each follow-up visit

Range of Flexion	Number of patients		
	At 1 1/2 month	At 3rd month	At 6th month
< 60°	22	3	2
60° – 90°	13	31	12
90° – 120°	0	1	21
120° – 180°	0	0	0
Total	35	35	35

At one and a half month, 68.5% (24 out of 35) of patients had abduction < 60. At 3 months, 60% (21 out of 35) in the range 60-90. At 6 months 60% (22 out of 35) in the range 90-120 as shown in Table II. Thus we have found that abduction improved over the study duration and was found to be significant (P value <0.001 Fisher’s exact test used).

**Table II:** Number of patients in defined abduction range at each follow-up visit

Range of Abduction	Number of patients for Abduction		
	At 1 1/2 month	At 3rd month	At 6th month
< 60	24	8	1
60 – 90	11	21	12
90 – 120	0	6	22
120 – 180	0	0	0
Total	35	35	35

For external rotation at one and a half month, 19 out of 35 patients had external rotation in the range of 30-60º and at 3 months, 28 out of 35 patients had external rotation in the range of 30-60º and lastly at 6 months, 30 out of 35 had external rotation >30º as shown in Table III. Thus we found increasing number of patients having improved range of external rotation over our study duration of six months and were found to be significant (P value <0.001 Fischer’s exact test used).

**Table III:** Number of patients in defined external rotation range at each follow-up visit

External Rotation (degrees)	Number of patients at each follow-up visits		
	At 1 1/2 month	At 3rd month	At 6th month
< 30	16	7	5
30 – 60	19	28	18
60 – 90	0	0	12
Total	35	35	35

For internal rotation at one and a half month, 20 out of 35 patients had internal rotation in the range of 30-60º and at 3 months 27 out of 35 patients had internal rotation in the range of 30-60º and at 6 months 23 out of 35 had internal rotation of 60-90º as shown in Table IV. Thus we found increasing number of patients had improved range of internal rotation over our study duration of six months and was found to be significant ( P value <0.001 Fisher’s exact test used).

**Table IV:** Number of patients in defined internal rotation range at each follow-up visit

Internal Rotation (degrees)	Number of patients at each follow-up visits		
	At 1 1/2 month	At 3rd month	At 6th month
< 30	15	8	6
30 – 60	20	27	6
60 – 90	0	0	23
Total	35	35	35

The absolute constant score of the study population increases at three months and six months as shown in Table IV. This was found to be significant (P value <0.001 Fisher’s exact test used). The Constant score at one and half, three months and six months correlated with the age of the patient and was significant ( Pearson’s correlation coefficient “r” = 0.248,0.399 and 0.305, respectively, p values <0.001 for all the 3). The Constant score decreased with increasing age which signified a poorer outcome in the elderly age group as shown in Table VI.

**Table V:** Distribution of absolute constant score post-operatively at each follow-up visit

Absolute Constant Score	Number of patients		
	At 1 1/2 month (%)	At 3rd month (%)	At 6th month (%)
0 – 25	1 (2.86%)	0	0
26 – 50	34 (97%)	35 (100%)	16 (45.71%)
51 – 75	0	0	19 (54.29%)
76 – 100	0	0	0
Total	35	35	35

**Table VI:** Absolute constant score vs age of patient

Absolute Constant Score	Age group				
	18 - 50 (n=19)		> 50 (n=16)		P-value
	Mean	SD	Mean	SD	
At 1 1/2 month	37.52	5.86	35.62	4.83	< 0.001
At 3rd month	44.89	2.90	42.81	3.27	< 0.001
At 6th month	52.89	5.65	50.5	7.69	< 0.001

**Fig. 4:**
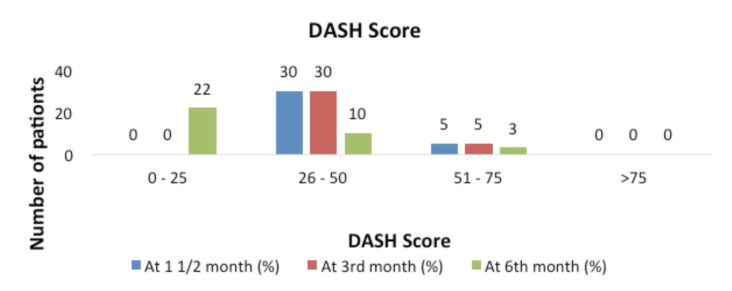
Distribution of DASH score

The data shows a decrease in the DASH score from three to six months in the study population and corresponded to a better outcome at 6 months as shown in Fig. 4. This was found to be significant (P value <0.001 Fischer’s exact test used).

**Fig. 5:**
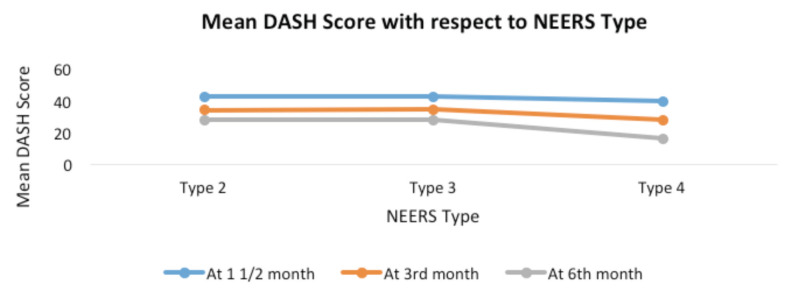
Mean DASH score vs type of fracture.

We found that all three types of proximal humerus fractures show significant improvement in the mean DASH score over our study period of six months as shown in Fig. 5 and was found to be significant (P value <0.001 kruskar wallis and mann whitney test used). The DASH score at one and half, three months and six months does correlated with the age of the patient (Pearson’s correlation coefficient “r” = 0.53 ,0.45 and 0.45, respectively, p values <0.001 for all the 3). The DASH score increased with increasing age which signified a poorer outcome in the elderly age group as shown in Table VII.

**Table VII:** Mean DASH score vs age of patient

DASH Score at	Age group				
	18 - 50 (n=19)		> 50 (n=16)		P-value
	Mean	SD	Mean	SD	
At 1 1/2 month	41.92	5.92	43.20	7.98	< 0.001
At 3rd month	33.67	9.53	34.58	11.80	< 0.001
At 6th month	27.01	11.67	27.69	14.47	< 0.001

**Fig. 6:**
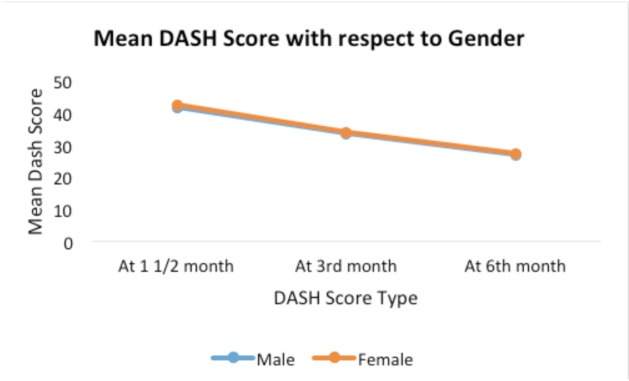
Mean DASH score vs gender.

The mean DASH score in females was higher than in males at the one and half, third and sixth month as shown in Fig. 6. But only one and half months difference was found to be significant (p-value < 0.05). This indicated a poorer functional outcome in females than in males with respect to DASH score. Out of the 35 cases two had complications. One had implant failure (Neer’s type 3, 60 year old female) and one had varus collapse (Neer’s type 3, 45-year-old male) as shown in Fig. 1.

## Discussion

Our results demonstrated several benefits of using proximal humerus locking plates.

In our study of a series of 35 patients admitted in a single center selected in a non-randomised manner and operated using proximal humerus locking plates for displaced and unstable proximal humerus fractures the results in most of the patients was found to be good as compared to comparable studies in the field.

The outcome was found to be better in younger patients and those with less complex fractures. There was a significant improvement in the functional outcome scores over the period of six months, which was found in other comparable studies.

The rate of complication was higher in elderly patients due to osteopenic bone and those with more complex fractures. The rate of fracture union especially in cases with good reduction and complication rates were within acceptable limits in our study.

Most of the complications were due to inaccurate reduction or improper operative technique. The results of management of proximal humeral fractures with the PHILOS plate were equally good in all the patients but in particular the functional outcome was better in younger patients.

In elderly patients, proximal humeral fractures often resulted from low-energy injuries to osteoporotic bone. As most of them were displaced 2- and 3-part fractures, non-operative treatment for these fractures was more likely given poor functional outcomes and a higher complication rate compared to operative management. Hence, surgical management is often a good option for such patients compared with conventional plate fixation, the locking plate system gives a better anatomic reduction with angular stability in osteoporotic bone and allows early mobilisation, which minimises post-operative complications and improves post-operative shoulder range. Therefore, locking plate fixation is recommended for the treatment of unstable proximal humerus fractures, especially those associated with osteoporosis.

As in the other studies, we took the DASH and Constant scores to determine the functional shoulder outcomes. The DASH score is a subjective assessment in which the functional results are reported by patients, whereas the Constant score is an objective assessment in which the functional results are determined by clinicians.

## Conclusion

Due to angular stability and effective maintenance of the intra-operative fracture reduction during follow-up period, early post-operative mobilisation is possible which helps the patient to attain better shoulder range of motion and return to activity faster. Loss of reduction was rarely seen compared with other implants. Patients older than 50 years had a higher chance of developing any type of complication.
